# Comparing the Japanese Version of the Ocular Surface Disease Index and Dry Eye-Related Quality-of-Life Score for Dry Eye Symptom Assessment

**DOI:** 10.3390/diagnostics10040203

**Published:** 2020-04-07

**Authors:** Takenori Inomata, Masahiro Nakamura, Masao Iwagami, Akie Midorikawa-Inomata, Yuichi Okumura, Keiichi Fujimoto, Nanami Iwata, Atsuko Eguchi, Hurramhon Shokirova, Maria Miura, Kenta Fujio, Ken Nagino, Shuko Nojiri, Akira Murakami

**Affiliations:** 1 Department of Ophthalmology, Juntendo University Faculty of Medicine, Tokyo 1130033, Japan; amurak@juntendo.ac.jp; 2 Department of Strategic Operating Room Management and Improvement, Juntendo University Faculty of Medicine, Tokyo 1130033, Japan; 3 Department of Hospital Administration, Juntendo University Graduate School of Medicine, Tokyo 1130033, Japan; ak-inomata@juntendo.ac.jp (A.M.-I.); a-eguchi@juntendo.ac.jp (A.E.); k-nagino@juntendo.ac.jp (K.N.); 4 Precision Health, Department of Bioengineering, Graduate School of Engineering, The University of Tokyo, Tokyo 1138656, Japan; masahiro-nakamura@umin.ac.jp; 5 Department of Health Services Research, University of Tsukuba, Ibaraki 3050006, Japan; iwagami-tky@umin.ac.jp; 6 Department of Ophthalmology, Juntendo University Graduate School of Medicine, Tokyo 1130033, Japan; y-okumura@juntendo.ac.jp (Y.O.); k-fujimoto@juntendo.ac.jp (K.F.); n-iwata@juntendo.ac.jp (N.I.); h-shokirova@juntendo.ac.jp (H.S.); maria-k@juntendo.ac.jp (M.M.); k.fujio.zz@juntendo.ac.jp (K.F.); 7 Department of Medical Technology Center, Juntendo University Faculty of Medicine, Tokyo 1130033, Japan; s-nojiri@juntendo.ac.jp

**Keywords:** dry eye disease, ocular surface disease index, dry eye-related quality-of-life score, questionnaire, subjective symptom, japanese version of OSDI, J-OSDI

## Abstract

The aim of this study was to compare patient-reported symptoms of dry eye disease (DED) between the Japanese version of the Ocular Surface Disease Index (J-OSDI) and the Dry Eye-Related Quality-of-Life Score (DEQS). A total of 169 participants were enrolled between September 2017 and May 2018. Patients were administered the J-OSDI and DEQS questionnaires at their first (baseline) and follow-up visits to evaluate DED-related symptoms. The correlations between the J-OSDI total score and DEQS (Frequency and Degree) scores were evaluated using Pearson’s correlation coefficient, and their clinical differences were assessed using the Bland–Altman analysis. At the baseline visit, the J-OSDI score and DEQS (Frequency and Degree) were significantly correlated (*r* = 0.855, *r* = 0.897, respectively). Moreover, a significant correlation was found between the J-OSDI score and DEQS (Frequency and Degree) at the follow-up visit (*r* = 0.852, *r* = 0.888, respectively). The Bland–Altman analysis revealed a difference (bias) of 4.18 units at the baseline and 4.08 units at the follow-up between the scores of the two questionnaires. The J-OSDI and DEQS were significantly correlated with negligible score differences, suggesting that the J-OSDI can be reliably used for Japanese patients, allowing for cross-country comparisons.

## 1. Introduction

Dry eye disease (DED) is one of the most common eye disorders affecting 5–50% of the population, and it is becoming more prevalent due to the ageing population and the increase in digital work [1,2,3,4]. It has become clear from previous large-scale crowdsourced research on real-world data collected using the iPhone application “DryEyeRhythm” that many people with dry eye symptoms remain undiagnosed [4,5,6]. DED causes various symptoms affecting ocular and visual function that interfere with the quality of vision and reduce work productivity [7,8]. Therefore, it is very important to screen for the various subjective symptoms of DED and link them to treatment [9].

The assessment of subjective symptoms is the fundamental examination for the diagnosis of DED [10,11] and has received greater attention than before, especially because the 2016 Asia Dry Eye Society diagnostic criteria are only based on subjective symptoms and the tear film breakup time (TFBUT) [11]. It is desirable to quantitatively assess subjective DED symptoms using questionnaires because there are several symptoms, including dryness, irritation, decreased visual acuity, and photophobia.

Several questionnaires are used for the objective assessment of the subjective symptoms of DED, such as the Ocular Surface Disease Index (OSDI), Standard Patient Evaluation of Eye Dryness Questionnaire (SPEED), and McMonnies questionnaire [12,13,14,15]. However, the only questionnaire that has been validated in Japan is the Dry Eye-Related Quality-of-Life Score (DEQS) [13]. As the DEQS has only been validated in Japanese, the epidemiological status of DED cannot be directly compared between Japan and other countries using this instrument. In contrast, the OSDI is commonly used worldwide. The validity and reliability of the Japanese version of the OSDI (J-OSDI) were confirmed by Inomata et al. in 2019 [16], and it became possible to perform epidemiological and symptomatic comparisons of DED between Japan and other countries. The J-OSDI and DEQS are now available as validated dry eye questionnaires in Japan, but the purpose of both these dry eye questionnaires are different, and both questionnaires have pros and cons. In addition, the OSDI was developed in 1997 [12]; therefore, the questions may be older and less relevant than those of the DEQS, which was developed in 2013 [13]. Therefore, it is important to identify a quantitative method that can be used for assessing subjective symptoms of dry eye in Japan.

Here, we compared the J-OSDI and DEQS questionnaires with a clinic-based cohort of patients with DED.

## 2. Materials and Methods

### 2.1. Study Design and Participants

This cross-sectional observational study included 169 patients who had been previously diagnosed with DED, and they were recruited between September 2017 and October 2018 from the Ocular Surface Unit of Juntendo University Hospital, Department of Ophthalmology, Tokyo, Japan. Written informed consent was obtained from all participants. This clinical study was approved by the Juntendo University Hospital Independent Ethics Committee (approval number, 17-088, 28 July 2017) and adhered to the tenets of the Declaration of Helsinki.

### 2.2. Inclusion and Exclusion Criteria

Patients were selected on the basis of the following criteria at the first (baseline) visit: symptoms of dry eye (dryness, burning, irritation, grittiness, foreign body sensation, or fluctuating vision) and/or decreased TFBUT (≤5 s). We excluded patients with decreased best corrected visual acuity (BCVA) (lower than 20/20), active infection, a history of eye lid disorders, ptosis, Parkinson’s disease, any other diseases affecting blinking including blepharospasm and thyroid eye disease, ocular surface surgeries, penetrating keratoplasty, eyelid surgeries, and hereditary corneal diseases.

### 2.3. Environmental Conditions

The temperature and humidity of the examination room were controlled at 26 °C in summer and 24 °C in winter with 50% relative humidity, according to the Guideline for the Design and Operation of Hospital Heating, Ventilation, and Air Conditioning Systems established by the Healthcare Engineering Association of Japan standard [17].

### 2.4. Dry Eye Symptom Assessment by the Questionnaires

Subjective symptoms were collected using the J-OSDI and DEQS. The J-OSDI was validated for Japanese individuals by a previous study [16]. The J-OSDI questionnaire consists of 12 questions with three subscales: ocular symptoms, vision-related function, and environmental triggers in accordance with the original English version (Allegan, Inc., Irvine, CA) of the OSDI [12]. The questionnaire is presented in Table S1. Each patient rated symptoms on a 5-point scale from always (score 4) to never (score 0) for each question. The OSDI total score and each subscale are graded on a scale from 0 to 100. According to the OSDI total score, the patients were classified as normal (0–12 points) or as having mild (13–22 points), moderate (23–32 points), or severe (33–100 points) symptoms [18].

The DEQS was used to assess the severity of dry eye-associated symptoms and the multifaceted effects of DED on the patients’ daily lives [13]. The DEQS includes two subscales, which first assess the frequency of the symptoms and disability (0 to 4) and then assess symptom degree (1 to 4). The questionnaire is presented in Table S2. The DEQS (Frequency and Degree) scores were calculated with the following formula: score = [sum of the Frequency or Degree scores for all questions answered] × 25/(total number of questions answered). The score derived from this questionnaire is a subjective measurement of DED symptoms, where 0 indicates the best possible score (no symptoms) and 100 indicates the worst possible score (maximum symptoms).

### 2.5. Dry Eye Disease Diagnosis and Classification

Both eyes of all patients underwent a complete ophthalmic evaluation, including measurement of BCVA, noncontact intraocular pressure, and subjective symptoms. TFBUT, cornea fluorescence staining (CFS) for keratoconjunctival vital staining, maximum blink interval (MBI), and Schirmer’s test I for reflex tear production, were collected for both eyes. The worst TFBUT, CFS, and Schirmer’s test I values were recorded as the subjective symptoms affected both eyes. The average MBI data were calculated from both eyes according to a previous study [19].

### 2.6. Clinical Assessments

TFBUT and kerato-CFS were assessed with fluorescein sodium (Fluorescence Ocular Examination Test Paper, Ayumi Pharmaceutical Co., Tokyo, Japan) staining. We performed TFBUT, CFS, and MBI measurements and subsequently Schirmer’s test I.

TFBUT was measured using a fluorescein dye according to the standard method [11]. In order to minimize the effect on tear volume and TFBUT, a small quantity of dye was administered with a wetted fluorescein strip. After the dye was instilled, the subject was instructed to blink three times to ensure adequate mixing of the dye with the tears. The interval between the last blink and the appearance of the first dark spot on the cornea was measured with a stopwatch. The mean value of the three measurements was used. The cut-off value of TBUT ≤ 5 s was used to diagnose DED [11]. CFS was graded according to the van Bijsterveld grading system [20], dividing the ocular surface into three zones: the nasal bulbar conjunctiva, temporal bulbar conjunctiva, and cornea. Each zone was evaluated on a scale of 0 to 3, with 0 indicating no staining and 3 indicating confluent staining. The maximum possible score is 9. The length of time that subjects could keep their eyes open before blinking during each trial was termed the MBI [19]. We calculated the MBI twice by a stopwatch under slit-lamp microscopy with the light turned off to avoid dazzling the patient. The MBI was set to 30 if the MBI exceeded 30 s. Following all other examinations, Schirmer’s test I was performed without topical anesthesia. Schirmer’s test strips were placed at the outer one-third of the temporal lower conjunctival fornix for 5 min. The strips were then removed, and the length of the dampened filter paper (in mm) was recorded.

### 2.7. Sample Size Calculation

Setting an α error of 0.05 and power (1-β) of 0.8, the required sample sizes for identifying statistically significant correlation coefficients of ≥ 0.3, ≥ 0.5, and ≥ 0.7 were estimated to be 85, 29, and 13 [21], respectively.

### 2.8. Statistical Analyses

In order to compare the characteristics of the participants, the two-tailed paired *t*-test was used for continuous variables. Pearson’s product–moment correlation coefficient was used to determine the correlation between the J-OSDI and DEQS (Frequency and Degree). A heatmap was constructed using the heatmap function of the seaborn module (ver. 0.9.0, Python 3). Bland–Altman analysis [22] was conducted to provide indication of the systematic random error, heteroscedasticity of the data, and 95% limits of agreements (LoA) of the two questionnaires. The variables used for the Bland–Altman analysis were the J-OSDI total score and the DEQS (Frequency and Degree) at the baseline and follow-up visits. Data are presented as means ± standard deviation (SD) or proportion (%). Statistical analyses were performed using STATA version 15 (Stata Corp, College Station, TX). *p* < 0.05 was considered significant.

## 3. Results

### 3.1. Participant Characteristics

A total of 169 patients were evaluated at two different visits (baseline and follow-up); they responded to the questionnaire, completed the examination, and were found eligible for analysis. Patient background characteristics are shown in Table 1. The average age at the baseline visit was 61.7 ± 14.1 years, and 84.6% (143/169) of the participants were women. The mean interval between the baseline and follow-up visits was 135.1 ± 65.4 days (median, 175 days; range, 14–410 days).

### 3.2. Scores of the J-OSDI and DEQS (Frequency and Degree)

The J-OSDI and DEQS scores are shown in Tables S1 and S2, respectively. At the baseline visit, the mean J-OSDI total score was 31.6 ± 22.3 (median, 29.2; range, 0–90), while the mean DEQS (Frequency) was 27.4 ± 22.6 (median, 20.0; range, 0–96.7) and mean DEQS (Degree) was 27.6 ± 22.6 (median, 20.0; range, 0–96.7). At the follow-up visit, the mean J-OSDI total score was 32.1 ± 22.6, while the mean DEQS (Frequency) was 27.3 ± 22.3 (median, 21.7; range, 0–93.3) and mean DEQS (Degree) was 28.7 ± 22.4 (median, 23.3; range, 0–98.3). On the basis of the J-OSDI total score, 41 (24.3%) participants reported normal (0–12), 53 (31.4%) reported mild to moderate (13–32), and 75 (44.4%) reported severe (33–100) dry eye symptoms.

### 3.3. Correlation between the J-OSDI and DEQS

**Figure 1:**
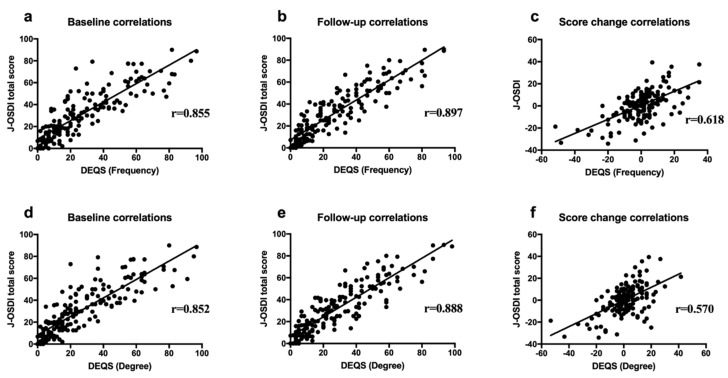
Correlation between the J-OSDI score and DEQS (Frequency and Degree) of patients with dry eye disease at the baseline and follow-up visits. Figure 1 shows the correlation between the J-OSDI total score and DEQS (Frequency) (**a**–**c**) and DEQS (Degree) (**d**–**f**) at the baseline and follow-up visits. The score changes between the baseline and follow-up visits were compared between the J-OSDI total score and DEQS (Frequency) (**a**–**c**) and DEQS (Degree) (**d**–**f**). J-OSDI: Japanese version of Ocular Surface Disease Index, DEQS: Dry Eye-Related Quality Score.

Figure 1 shows the correlation between the J-OSDI total score and the DEQS (Frequency and Degree). There were significant positive correlations between the J-OSDI total score and DEQS (Frequency) at the baseline (Figure 1a; *r* = 0.855, *p* < 0.001) and at the follow-up visit (Figure 1b; *r* = 0.897, *p* < 0.001). Changes in the J-OSDI total score and DEQS (Frequency) from the baseline to the follow-up visit are shown in Figure 1c; they were significantly positively correlated (*r* = 0.618, *p* < 0.001). In addition, there were significant positive correlations between the J-OSDI total score and DEQS (Degree) at baseline (Figure 1d; *r* = 0.852, *p* < 0.001) and at the follow-up visit (Figure 1e; *r* = 0.888, *p* < 0.001). Changes in the scores from the baseline to the follow-up visits for the J-OSDI total score and DEQS (Degree) are shown in Figure 1f, and they were significantly positively correlated (*r* = 0.570, *p* < 0.001).

Table 2 shows the correlation between the J-OSDI total score and DEQS (Frequency and Degree) based on the severity of DED symptoms. The J-OSDI total score and DEQS (Frequency and Degree) were positively correlated between the baseline and follow-up visits for all DED symptom severity categories; however, the mild to moderate subgroup of the J-OSDI total score had a relatively low correlation coefficient with DEQS scores at the follow-up visit.

**Figure 2:**
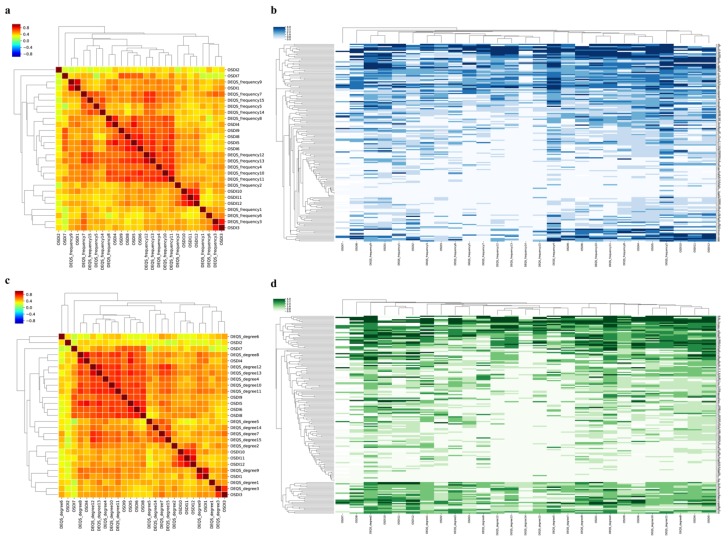
Correlation between the J-OSDI and DEQS at the baseline visit. Pearson’s correlation coefficients and scores between the J-OSDI and DEQS are shown in the heatmap as a color gradient. (**a**) Heatmap of the correlation between the J-OSDI and DEQS (Frequency) at the baseline visit. (**b**) Heatmap with clustering of the J-OSDI score and DEQS (Frequency) in all participants. (**c**) Heatmap of the correlation between the J-OSDI score and DEQS (Degree) at the baseline visit. (**d**) Heatmap with clustering of the J-OSDI score and DEQS (Degree) in all participants. Color scale bars: Correlation coefficients (**a**,**c**) and the 5-point scale for each question scores (**b**,**d**). Axis: each question from the OSDI and DEQS.

The heatmap shows the association between the J-OSDI and DEQS at the baseline visit (Figure 2) and follow-up visit (Figure S1). As seen in Figure 2a–d, the similar questions between the J-OSDI and DEQS (Frequency and Degree) were positively correlated; there were correlations between OSDI1 and DEQS9, OSDI3 and DEQS3, OSDI4 and DEQS8, OSDI6 and DEQS10, OSDI8 and DEQS11, and OSDI9 and DEQS11. The variation in the individual subjective symptoms of DED measured by the J-OSDI and DEQS is shown in Figure 2b,d.

### 3.4. Comparison of the J-OSDI Total Score and DEQS (Frequency and Degree)

**Figure 3:**
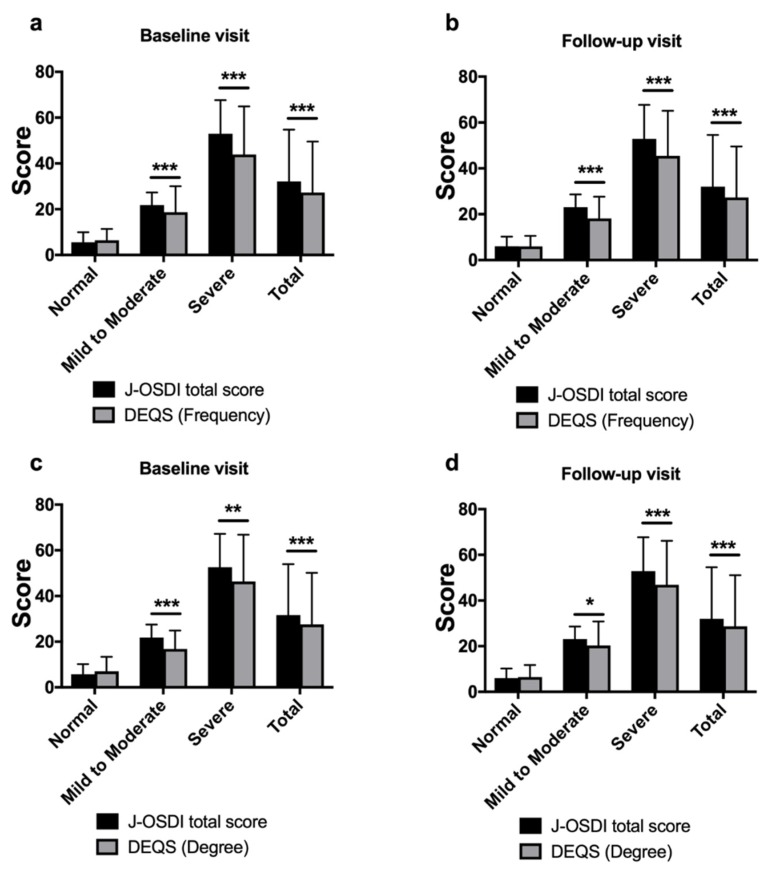
Comparison of the J-OSDI total score and DEQS (Frequency and Degree) of patients with dry eye disease based on the J-OSDI subgroups; (**a**,**b**) show the comparison between the J-OSDI total score and DEQS (Frequency) based on the J-OSDI subgroups at baseline (**a**) and follow-up (**b**) visits, and (**c**,**d**) show the comparison between the J-OSDI total score and DEQS (Degree) based on the J-OSDI subgroups at baseline (**c**) and follow-up (**d**) visits. Data are considered statistically significant at * *p* < 0.05, ** *p* < 0.01, and *** *p* < 0.001.

Figure 3 shows the comparison of the J-OSDI total score and DEQS (Frequency and Degree) at the baseline and follow-up visits. At the baseline visit, the J-OSDI total score was significantly higher than the DEQS (Frequency) for mild to moderate symptoms (J-OSDI, 21.8 ± 5.7 vs. DEQS, 16.5 ± 8.6; *p* < 0.001), severe symptoms (J-OSDI, 52.6 ± 14.6 vs. DEQS, 46.0 ± 20.8; *p* < 0.001), and total score (J-OSDI 31.6 ± 22.4, DEQS 27.4 ± 22.6; *p* < 0.001). At the follow-up visit, the J-OSDI total score was also significantly higher than the DEQS (Frequency) for mild to moderate symptoms (J-OSDI, 23.1 ± 5.6 vs. DEQS, 18.2 ± 9.5; *p* < 0.001), severe symptoms (J-OSDI, 52.9 ± 14.8 vs. DEQS, 45.5 ± 19.6; *p* < 0.001), and total score (J-OSDI, 32.1 ± 22.6 vs. DEQS, 27.3 ± 22.3; *p* < 0.001).

Similarly, at the baseline visit, the J-OSDI total score was significantly higher than the DEQS (Degree) for mild to moderate symptoms (J-OSDI, 21.8 ± 5.7 vs. DEQS, 16.9 ± 8.0; *p* < 0.001), severe symptoms (J-OSDI, 52.6 ± 14.6 vs. DEQS, 46.4 ± 20.5; *p* = 0.001), and total score (J-OSDI 31.6 ± 22.4, DEQS 27.6 ± 22.6; *p* < 0.001). At the follow-up visit, the J-OSDI total score was also significantly higher than the DEQS (Degree) for mild to moderate symptoms (J-OSDI, 23.1 ± 5.6 vs. DEQS, 20.3 ± 10.5; *p* = 0.038), severe symptoms (J-OSDI, 52.9 ± 14.8 vs. DEQS, 46.9 ± 19.2; *p* < 0.001), and total score (J-OSDI, 32.1 ± 22.6 vs. DEQS, 28.7 ± 22.4; *p* < 0.001).

### 3.5. Bland–Altman Analysis

**Figure 4:**
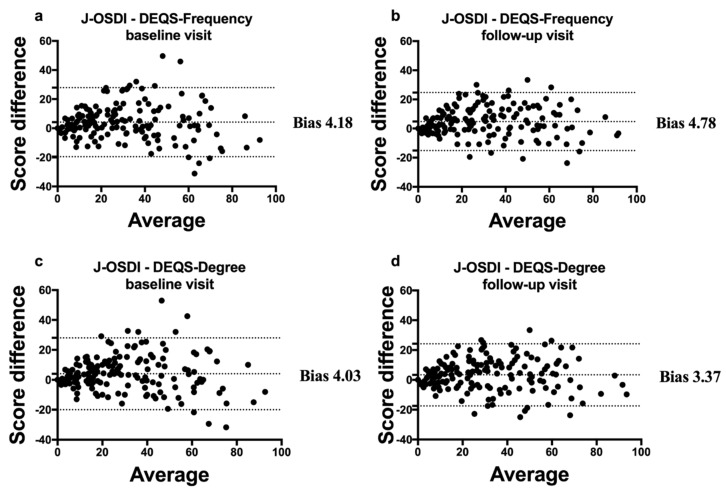
Bland–Altman plot for the J-OSDI total score and DEQS (Frequency and Degree). The x-axis indicates the average of the two questionnaire scores, and the y-axis indicates the difference between the two questionnaire scores (J-OSDI and DEQS). The central line indicates the mean difference (bias) between the scores from the two questionnaires, whereas the superior and inferior lines depict the intervals, which include the 95% limits of agreement. Differences between the J-OSDI and DEQS (Frequency) at the baseline visit (**a**) and follow-up visit (**b**). Differences between the J-OSDI and DEQS (Degree) at the baseline visit (**c**) and follow-up visit (**d**).

Bland–Altman analysis for the clinical agreement between the J-OSDI and DEQS (Frequency) revealed a clinical difference (bias) with a 95% LoA of 4.18 (−19.5–27.9) units at baseline and 4.78 (−15.1–24.7) units at the follow-up visit, while analysis between the J-OSDI and DEQS (Degree) revealed a clinical difference (bias) of 4.03 (−19.9–28.0) units at baseline and 3.37 (−17.5–24.2) units at the follow-up visit (Figure 4).

## 4. Discussion

DED consists of a variety of subjective symptoms. Therefore, it is important to objectively monitor the subjective symptoms for DED screening and treatment evaluation [4,5,6]. In this study, we compared two questionnaires for dry eye subjective symptoms, the J-OSDI and DEQS, which can be only used in Japan. This study identified that both the J-OSDI and DEQS were useful for evaluating subjective symptoms of DED as their scores were correlated, and there was limited bias between them. Therefore, the J-OSDI can be used in Japan to perform epidemiological comparisons with the results of clinical studies conducted in other countries.

In this study, the J-OSDI and DEQS, which are validated for use in Japan, were compared in patients with DED, and we confirmed the concurrent signs and symptoms of the disease. The J-OSDI total scores at the baseline and follow-up visits were well correlated with both DEQS (Frequency) and DEQS (Degree) (Figure 1, Figure 2, and Figure S1). Likewise, the correlation between changes in the J-OSDI and DEQS (Frequency and Degree) from the baseline to follow-up visits remained significant (Figure 1c,f). In addition, the J-OSDI total scores were well correlated with those of the DEQS (Frequency and Degree) when the J-OSDI total score was classified based on the severity of DED (Table 2). These data indicate that both the J-OSDI and DEQS are useful for assessing DED symptoms in Japan.

As shown in Figure 2, the J-OSDI total score tended to be higher than the DEQS (Frequency and Degree). This tendency became more pronounced as the subjective symptoms of DED became more severe. This difference in scores may be due to differences in the number of questions between the J-OSDI (12 questions) and DEQS (15 questions); therefore, the gravity of each question may differ between the two questionnaires. In addition, the J-OSDI can assess environmental factors and the DED effect on night driving, and these items may have contributed to the score difference between the two questionnaires for severe DED. However, the Bland–Altman analysis showed limited differences between the J-OSDI total score and DEQS (Frequency and Degree) at the baseline and follow-up visits. Moreover, there were a few outliers affecting the 95% LoA. These results suggest that there is limited difference between the J-OSDI and DEQS and that both are useful for assessing the subjective symptoms of DED. However, although there are few differences between the two questionnaires, the two should not be used interchangeably when monitoring symptoms of DED in daily clinical practice.

Table 3 shows the comparison between the J-OSDI and DEQS features. Both questionnaires could evaluate the subjective symptoms of DED; the J-OSDI evaluates the visual function-related subjective DED symptoms, whereas the DEQS evaluates dry eye symptoms in daily life. Therefore, it is recommended to use each questionnaire according to the purpose of the evaluation. The DEQS was developed in 2013 [13], more recently than the OSDI, which was developed in 2000 [12]. As shown in Tables S1 and S2, the DEQS may be more relevant today as it includes questions pertaining to depressive symptoms and eye issues stemming from looking at a cell phone screen [23,24]. However, the J-OSDI can be used to evaluate the effect of environmental factors not included in the DEQS. Regarding the number of questions, the DEQS may be somewhat cumbersome for respondents because it requires responding to up to 30 questions compared to 12 questions in the J-OSDI. However, while the J-OSDI only asks questions on frequency, the DEQS can quantify both frequency and degree. Scores are graded from 0–100 in both questionnaires, but the DEQS cannot estimate severity by score unlike the J-OSDI. As for the validity by language, the J-OSDI has been confirmed to be valid in both Japanese and English [16], whereas the DEQS has only been validated in Japanese. As the OSDI is used worldwide, it can be used for comparisons between Japan and other countries.

This study had several limitations. First, it may have had a selection bias as it was conducted at a single university hospital in Japan, and there were more female participants, probably because DED primarily affects women [25]. Second, recall bias may have been present in this questionnaire survey, resulting in over-reporting of the subjective symptoms of DED. Finally, important unmeasured DED-related factors, including the use of systemic medications, depression, and anxiety, may have affected the results. In addition, this study did not examine seasonal effects as it had a cross-sectional design.

In conclusion, data collected from the J-OSDI and DEQS questionnaires were significantly correlated with negligible score differences. However, as the J-OSDI total score tended to be higher than the DEQS (Frequency and Degree), the two should not be used interchangeably when monitoring DED symptoms in daily clinical practice. It is recommended to use each questionnaire according to the purpose of the evaluation.

## Figures and Tables

**Table 1:** The characteristics of study participants.

Characteristics	Baseline	Follow-up	*p* Value
	*n* = 169	*n* = 169	
Age, years ± SD	61.7 ± 14.1		-
Sex, female (%)	143 (84.6)		-
BCVA, logMAR ± SD	−0.066 ± 0.025	−0.062 ± 0.031	** 0.004
IOP, mmHg ± SD	13.9 ± 2.9	13.5 ± 2.7	0.085
TFBUT, second ± SD	1.6 ± 1.6	1.6 ± 1.1	0.716
CFS, 0–9 ± SD	3.3 ± 2.6	3.5 ± 2.8	0.180
Schirmer’s test I, mm ± SD	4.8 ± 5.3	4.0 ± 4.9	0.785
MBI, second ± SD	11.4 ± 7.1	11.5 ± 7.1	0.866

*p* Values were determined with Student’s *t*-tests and two-tailed *t*-test for continuous variables. BCVA: best corrected visual acuity, IOP: intraocular pressure, TFBUT: tear film breakup time, CFS: corneal fluorescein staining. MBI: maximum blink interval. Data are considered statistically significant at ** *p* < 0.01.

**Table 2:** Correlation between the J-OSDI total score and DEQS (Frequency and Degree) based on the J-OSDI subgroups at baseline.

	Baseline		Follow-up	
	DEQS			
J-OSDI, 0–100	Frequency	Degree	Frequency	Degree
Normal (0–12)	0.662	0.688	0.606	0.531
Mild to moderate (13–32)	0.665	0.462	0.358	0.378
Severe (33–100)	0.628	0.609	0.785	0.749

**Table 3:** Comparison of the J-OSDI and DEQS features.

	J-OSDI	DEQS
Purpose	Symptoms of ocular irritation consistent with DED and their impact on vision-related functioning	Symptoms and their effect on daily life
Development, year	2000	2013
Questions, number	12	30 (Frequency and Degree)
Score	0–100	0–100
Cut-off value, score	≥ 13	> 15
Severity classification	+	−
Validation in Japanese	+	+
Validation in English	+	−

DED: dry eye disease.

## Supplementary Materials

The following are available online at https://www.mdpi.com/2075-4418/10/4/203/s1, Figure S1. The correlation between the J-OSDI and DEQS at the follow-up visit; Table S1. J-OSDI score at the baseline and follow-up visits; Table S2. DEQS at the baseline and follow-up visits.
