# Supplementary material for: Comparing the Japanese Version of the Ocular Surface Disease Index and Dry Eye-Related Quality-of-Life Score for Dry Eye Symptom Assessment
Source: Diagnostics (Basel). 2020 Apr 7;10(4):203. doi: 10.3390/diagnostics10040203 (PMC7235869; doi:10.3390/diagnostics10040203)
Supplement: Supplementary file 1 [file diagnostics-10-00203-s001.pdf]

Title: Comparing the Japanese Version of the Ocular Surface Disease Index and Dry Eye-related Quality-of-Life Score for Dry Eye Symptom Assessment

Authors: *Takenori Inomata[1,2,3] * MD, PhD, MBA, Masahiro Nakamura[4] MD, PhD, Masao Iwagami[5] MD, MPH, MSc, PhD, Akie Midorikawa-Inomata[3] RN, MPH, Yuichi Okumura[6] MD, Keiichi Fujimoto[6] MD, Nanami Iwata[6], Atsuko Eguchi[3], Hurramoy Shokirova[6] MD, Maria Miura[6] MD, Kenta Fujio[6] MD, Ken Nagino[3], Shuko Nojiri[7] MPH, PhD, and Akira Murakami[1,6,] MD, PhD.*

Supplementary Information

Table S1. J-OSDI score at the baseline and follow-up visits

Visits	Baseline	Follow-up	
Item	n = 169	n = 169	P value
J-OSDI total score, 0–100	31.6 ± 22.3	32.1 ± 22.6	0.649
Ocular symptoms, 0–100	30.3 ± 21.6	30.6 ± 21.6	0.741
Eyes that are sensitive to light?	1.38 ± 1.30	1.34 ± 1.20	0.632
Eyes that feel gritty?	0.88 ± 1.01	1.02± 1.01	0.572
Painful or sore eyes?	0.80 ± 0.96	0.88 ± 91.01	0.572
Blurred vision?	1.43 ± 1.17	1.45 ± 1.23	0.781
Poor vision?	1.57 ± 1.23	1.50 ± 1.27	0.164
Vision-related function, 0–100	30.0 ± 27.6	30.3 ± 26.2	0.817
Reading?	1.36 ± 1.32	1.34 ± 1.24	0.738
Driving at night?	0.93 ± 1.31	0.95 ± 1.20	0.647
Working with a computer or bank machine (ATM)?	1.36 ± 1.36	1.32 ± 1.28	0.890

Watching TV?	1.07 ± 1.12	1.10 ± 1.11	0.690
Environmental triggers, 0–100	33.9 ± 30.8	34.5 ± 29.4	0.684
Windy conditions?	1.49 ± 1.36	1.49 ± 1.30	0.643
Places or areas with low humidity (very dry)?	1.22 ± 1.26	1.37 ± 1.19	0.261
Areas that are air conditioned?	1.52 ± 1.37	1.57 ± 1.28	0.551

J-OSDI: Japanese version of the Ocular Surface Disease Index. P values were determined using Student's t-tests. Data are considered statistically significant at $P < 0.05$, $P < 0.01$, and $P < 0.001$.

Table S2. DEQS at the baseline and follow-up visits

	Frequency			Degree		
Visits	Baseline	Follow-up		Baseline	Follow-up	
Item	n = 169		P value	n = 169		P value
DEQS summary score, 0–100	27.4 ± 22.6	27.3 ± 22.3	0.879	27.6 ± 22.6	28.7 ± 22.4	0.237
Bothersome Ocular Symptoms, 0–100	30.9 ± 23.2	30.8 ± 22.2	0.898	31.9 ± 22.9	33.1 ± 22.8	0.272
Foreign body sensation	1.18 ± 1.2	1.22 ± 1.2	0.596	1.31 ± 1.2	1.43 ± 1.3	0.143
Dry sensation in the eyes	1.98 ± 1.4	1.91 ± 1.3	0.331	1.88 ± 1.3	1.88 ± 1.2	0.936
Painful or sore eyes	0.95 ± 1.2	0.95 ± 1.1	0.933	1.12 ± 1.3	1.24 ± 1.3	0.348
Ocular fatigue	1.76 ± 1.4	1.72 ± 1.3	0.583	1.69 ± 1.3	1.70 ± 1.3	0.800
Heavy sensation in the eyelids	0.77 ± 1.2	0.82 ± 1.2	0.521	0.80 ± 1.2	0.85 ± 1.1	0.554
Redness in the eyes	0.80 ± 1.1	0.80 ± 1.2	0.999	0.86 ± 1.1	0.85 ± 1.2	0.887
Impact on Daily Life, 0–100	24.7 ± 24.0	24.9 ± 23.9	0.898	24.3 ± 23.9	25.7 ± 23.8	0.213
Difficulty opening the eyes	0.80 ± 1.1	0.90 ± 1.2	0.254	0.86 ± 1.2	0.98 ± 1.2	0.159
Sensitivity to bright light	1.29 ± 1.3	1.28 ± 1.2	0.743	1.31 ± 1.2	1.30 ± 1.3	0.775
Problems with the eyes when reading	1.42 ± 1.3	1.35 ± 1.3	0.238	1.29 ± 1.2	1.31 ± 1.2	0.794
Problems with the eyes when watching television or looking at a computer or cell phone	1.29 ± 1.4	1.22 ± 1.3	0.324	1.19 ± 1.2	1.24 ± 1.3	0.583

Feeling distracted because of eye symptoms	1.29 ± 1.3	1.37 ± 1.3	0.332	1.23 ± 1.2	1.36 ± 1.3	0.096
Eye symptoms after work	0.95 ± 1.2	0.98 ± 1.2	0.872	0.93 ± 1.2	1.03 ± 1.2	0.319
Eye symptoms affect work	0.85 ± 1.2	0.88 ± 1.2	0.664	0.90 ± 1.3	0.95 ± 1.2	0.616
Reluctant to go out because of eye symptoms	0.33 ± 0.8	0.28 ± 0.8	0.367	0.33 ± 0.8	0.35 ± 0.9	0.762
Feeling depressed because of eye symptoms	0.77 ± 1.2	0.70 ± 1.1	0.238	0.80 ± 1.2	0.75 ± 1.1	0.351

DEQS; the Dry Eye-Related Quality-of-Life Score. P values were determined using Student’s t-tests. Data are considered statistically significant at $P < 0.05$.

Figure S1. The correlation between the J-OSDI and DEQS scores at the follow-up visit. Pearson's correlation coefficients and scores between the J-OSDI and DEQS are shown in the heatmap as a colour gradient. Heatmap of the correlation between the J-OSDI and DEQS–Frequency at the follow-up visit (a). Heatmap with clustering of the J-OSDI scores and DEQS–Frequency in all participants (b). Heatmap of the correlation between the J-OSDI and DEQS–Degree at the follow-up visit (c). Heatmap with clustering of the J-OSDI scores and DEQS–Degree in all participants (d). Color scale bars: Correlation coefficients (a and c) and the 5-point scale for each question scores (b and d). Axis: each question from the OSDI and DEQS.

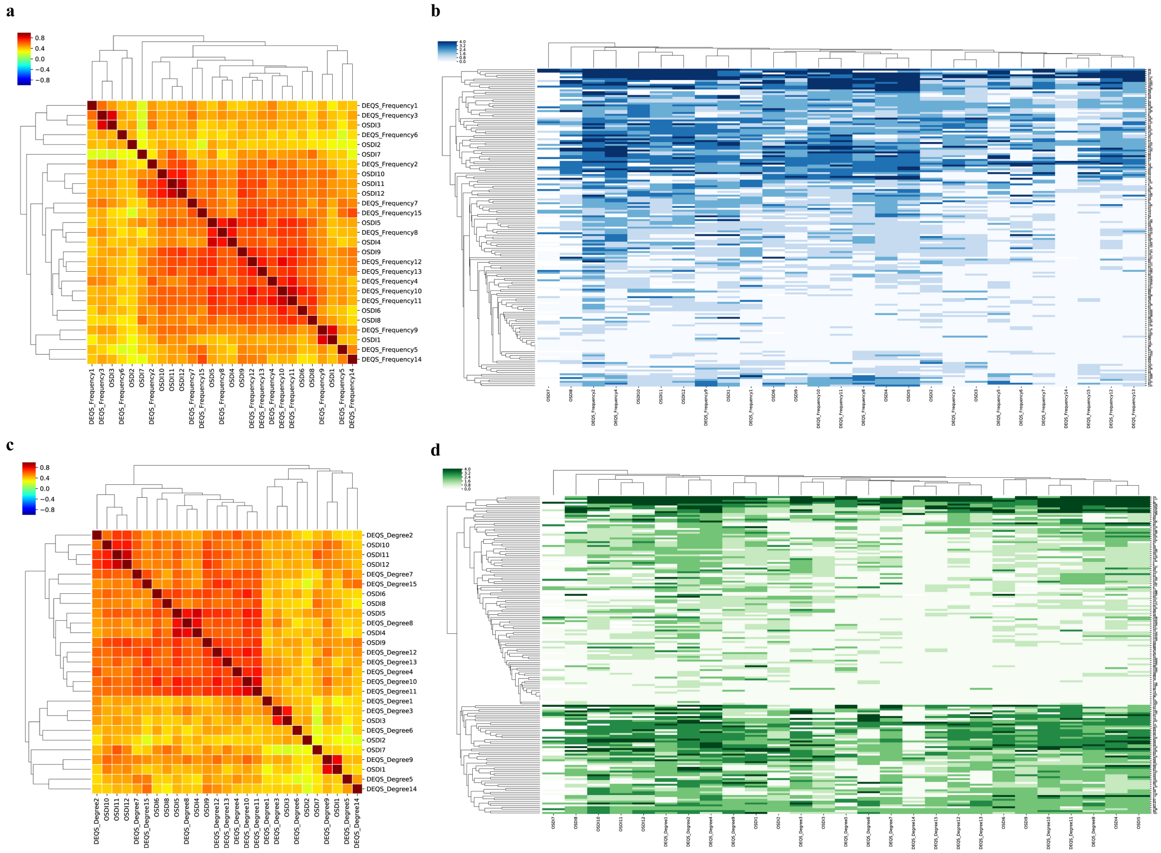
a
b
c
d
0.8
0.4
0.0
−0.4
−0.8
DEQS_Frequency1
DEQS_Frequency3
OSDI3
DEQS_Frequency6
OSDI2
OSDI7
DEQS_Frequency2
OSDI10
OSDI11
OSDI12
DEQS_Frequency7
DEQS_Frequency15
OSDI5
DEQS_Frequency8
OSDI4
OSDI9
DEQS_Frequency12
DEQS_Frequency13
DEQS_Frequency4
DEQS_Frequency10
DEQS_Frequency11
OSDI6
OSDI8
DEQS_Frequency9
OSDI1
DEQS_Frequency5
DEQS_Frequency14
DEQS_Degree2
OSDI10
OSDI11
OSDI12
DEQS_Degree7
DEQS_Degree15
OSDI6
OSDI8
OSDI5
DEQS_Degree8
OSDI4
OSDI9
DEQS_Degree12
DEQS_Degree13
DEQS_Degree4
DEQS_Degree10
DEQS_Degree11
DEQS_Degree1
DEQS_Degree3
OSDI3
DEQS_Degree6
OSDI2
OSDI7
DEQS_Degree9
OSDI1
DEQS_Degree5
DEQS_Degree14

27
